# Impact of multileaf collimator width on intraprostatic dose painting plans for dominant intraprostatic lesion of prostate cancer*

**DOI:** 10.1120/jacmp.v11i4.3193

**Published:** 2010-09-07

**Authors:** Eisuke Abe, Takashi Mizowaki, Yoshiki Norihisa, Yuuichirou Narita, Yukinori Matsuo, Masaru Narabayashi, Yasushi Nagata, Masahiro Hiraoka

**Affiliations:** ^1^ Division of Radiation Oncology, Department of Molecular Genetics Course for Molecular and Cellular Medicine, Graduate School of Medical and Dental Sciences, Niigata University Niigata Japan; ^2^ Department of Radiation Oncology and Image‐applied Therapy, Graduate School of Medicine Kyoto University Kyoto Japan; ^3^ Division of Radiation Oncology Hiroshima University Hospital Japan

**Keywords:** prostate cancer, dose painting, dominant intraprostatic lesions, multileaf collimator, leaf width

## Abstract

The aim of this study was to investigate the impact of multileaf collimator width (MLC‐W) on intraprostatic dose painting plans for prostate cancer.

Prostate cancer maps based on the histopathological findings were superimposed onto simulation CT images. Clinical target volume (CTV) 1 was defined as the prostate and the base of the seminal vesicles, and CTV2 was defined as the dominant intraprostatic lesions. Planning target volume (PTV) 1 and PTV2 were delineated by adding 5 mm margins to CTV1 and CTV2, respectively. For each case, two dose painting plans were created to deliver 74 Gy to PTV1 and 84 Gy to PTV2 with dynamic multileaf collimator technique using two different MLCs: m3 (MLC‐W: 3 mm) and Millennium (5 mm). Plans were evaluated by comparing the conformation number (CN), a quantity that defines the degree of conformality.

The CNs for plans using the m3 and Millennium were 0.68 and 0.67 for PTV1 and 0.59 and 0.58 for PTV2, respectively. The CNs tended to be higher for a thinner leaf width (p<0.05).

Dosimetric advantages associated with smaller leaves were observed. However, differences between 3 mm and 5 mm leaf width were relatively small, which suggested that 5 mm leaf width would be acceptable in dose painting plans for prostate cancer.

PACS numbers: 87.56.N‐, 87.56.nk, 87.55.D‐

## I. INTRODUCTION

Prostate cancer is a dose‐responsive neoplasm, and dose escalation to the prostate (plus seminal vesicles) has been successfully applied to improve local control and prostate‐specific antigen failure‐free survival rate.^(^ ^1^ ^–^ ^6^ ^)^ On the other hand, as the dose to the prostate is increased with the same radiation technique, the risk for late radiation adverse effects, particularly the incidence of rectal bleeding, also increases, because delivered doses to organs at risk (OARs) also increase.^(^ ^5^ ^,^ ^7^ ^,^ ^8^ ^)^

Intensity‐modulated radiotherapy (IMRT) has successfully achieved delivery of very high doses (76–86 Gy) while keeping the doses to OARs within an acceptable level with the aid of computer optimization and computer‐controlled IMRT. IMRT improves dose conformality to the target and reduces unnecessary radiation to surrounding OARs, which results in higher local control and lower late morbidity rates.^(^ ^6^ ^,^ ^9^ ^–^ ^11^ ^)^ However, it is estimated that doses even higher than 86 Gy are necessary to control high‐risk disease, especially cancers with high Gleason scores.^(^ ^6^ ^,^ ^12^ ^)^ Unfortunately, long‐term safety of delivering a dose higher than 90 Gy to the whole prostate plus seminal vesicles has not yet proven.

Prostate cancer typically has a multifocal distribution. There are usually a few dominant intraprostatic lesions (DILs), which are thought to require higher doses of radiation to be controlled; the majority of smaller lesions is expected to be controlled with conventional doses of 70–74 Gy, which are sufficient for good control of low‐risk disease. Fortunately, improved noninvasive prostate imaging modalities such as dynamic magnetic resonance imaging (MRI), magnetic resonance spectroscopy and diffusion‐weighted MRI, have the potential to successfully detect DILs. In addition, IMRT has the capability of dose painting,^(^ ^13^ ^)^ in which different doses can be delivered simultaneously to different areas within the target. This technique is also called simultaneous integrated boost irradiation.^(^ ^14^ ^)^ Therefore, a new strategy for treating prostate cancer by intraprostatic dose painting, in which very high doses are focused on DILs while other planning target volume (PTV) areas receive conventional doses, has been proposed.^(^ ^15^ ^–^ ^19^ ^)^

The multileaf collimator (MLC) has become a well‐accepted device in radiotherapy because it facilitates the delivery of irregularly‐shaped or intensity‐modulated treatment fields. Several studies on the dosimetric impact of collimator leaf width have been conducted and have confirmed the dosimetric advantage associated with smaller leaves in conformal radiotherapy and IMRT, mainly for cases with head‐and‐neck cancer.^(^ ^20^ ^–^ ^24^ ^)^ In dose painting plans for prostate cancer, it seems that smaller leaf width is more desirable because DILs are comparatively small. However, the gains of thinner leaves have not yet been quantified for dose painting plans in prostate cancer, especially in cases with dynamic multileaf collimator (DMLC) technique in which the resolution of dose gradient along the driving direction of leaves is fine. Moreover, given the relatively small size of DILs, the size of calculation grid may have a large influence on the plan evaluation.

In the present study, we evaluated the impact of the MLC width (MLC‐W) on dose distribution, and estimated how much leaf width is appropriate in clinical use in dose painting plans for prostate cancer delivered with DMLC technique.

## II. MATERIALS AND METHODS

### A. Creating prostate cancer models of DIL mapping

Prostate cancer maps were produced from pathological findings of whole mounted sections of resected prostate glands of seven patients with clinical stage T1–2N0M0 prostate cancer, who underwent radical prostatectomy at our institution. After radical prostatectomy, the resected specimens were fixed and cut perpendicularly to the urethra into thin multiple axial sections. The sections were examined microscopically by pathologists to produce a pathological map of cancer localization.

Seven patients with clinically localized prostate cancer who had been previously treated by IMRT in our institution were selected for this study. All planning CT scans were obtained by using a CT simulator (Light Speed RT; GE Healthcare, Waukesha, WI) with 2.5 mm slice thickness, without a gap. Patients were instructed to void the bladder and rectum about one to one‐and‐a‐half hours before the CT simulation, according to their individual urinary conditions. Patients were immobilized in the prone position using thermoplastic shells, with the combination of a vacuum pillow and a leg support. Prostate cancer localizations of the seven surgical cases were mapped on to the CT images of the arbitrarily selected seven IMRT cases.^(^ ^25^ ^)^

The positions of DILs were handwritten onto the CT images from prostate pathological maps, based on our mapping algorithm.^(^ ^26^ ^)^ In this study, cancer sites with a diameter larger than 5 mm were generally judged as DILs. These seven prostate cancer models of DIL mapping were used in the following planning studies.

All patients gave written informed consent about possibility of utilizing the patient's related materials for research purposes.

### B. Contouring of structures

Target delineations and treatment planning were performed with an Eclipse‐Helios system (Ver. 7.1.67; Varian Medical Systems, Palo Alto, CA). The prostate, rectal outer wall and bladder outer wall were contoured. Clinical target volume (CTV) 1 was defined as the prostate and the base of the seminal vesicles, and CTV2 was defined as the DILs. PTV1 and PTV2 were defined by adding a 5 mm margin to CTV1 and CTV2, respectively, in all directions. The rectal wall was generated from the rectal outer wall, using a wall‐extraction function with a wall thickness of 4 mm on every CT slice, from 10 mm below the apex of the prostate to 10 mm above the tips of the seminal vesicles. The bladder wall was generated from the bladder outer wall in the same manner as the rectal wall, with a wall thickness of 4 mm. The prostatic urethra was outlined from the prostate apex to the base.

### C. Intraprostatic dose painting planning protocol

For each case, intraprostatic dose painting plans were experimentally created using two different MLCs: m3 (BrainLAB, Feldkirchen, Germany; minimum MLC‐W: 3 mm) and Millennium (Varian Medical Systems; minimumMLC‐W:5mm). The BrainLAB m3 had three different leaf widths and a total of 26 pairs of leaves. The 14 innermost pairs of leaves projected to a 3 mm width at the isocenter; the intermediate six pairs, to a 4.5 mm width; and the outermost six pairs, to a 5.5 mm width. The Varian 120‐leaf Millennium MLC had two leaf widths, 5 mm (inner 40 pairs; used in this study) and 10 mm (outer 20 pairs).

All plans were designed for use with the 15 MV photon beam of a Clinac 2300C/D (Varian Medical Systems, Palo Alto, CA, USA). The dose distributions were calculated using a pencil‐beam convolution algorithm, with a calculation grid resolution of 2.5×2.5mm, to which the modified Batho heterogeneity correction was applied.

Intraprostatic dose painting plans were designed to deliver 74 Gy to PTV1 and 84 Gy to PTV2. To create the dose painting plans, a seven‐field DMLC technique was used. The beam arrangement was as follows: anterior (0°), right posterior oblique (40°, 75°), right anterior oblique (145°), left anterior oblique (215°), and left posterior oblique (285°, 320°) fields. Inverse treatment planning by computer optimization in the Helios System (Varian Medical Systems, Palo Alto, CA, USA) was used to create the IMRT plans. We repeated inverse optimization many times by adjusting parameters for optimization until the planning goals (Table 1) were satisfied.^(^ ^25^ ^)^

**Table 1:** Planning goals.

*Structure*		
PTV1 (Prescription dose: 74 Gy)	D95%	94.9%–95.1%
PTV2 (Prescription dose: 84 Gy)	D95%	94.9%–95.1%
Urethra		< 82 Gy
Rectal Wall	V40Gy	<60%
	V60Gy	<35%
	V70Gy	<25%
	V84Gy	<1%
Bladder Wall	V40Gy	<60%
	V70Gy	<35%

The maximum dose to PTV2 was to be identical within ± 0.1 Gy for each case among the corresponding plans with different MLCs.

The final dose distributions were recalculated with a calculation grid resolution of 1.25×1.25mm and 5×5mm.

### D. Comparison of treatment plans

The doses to the targets (PTV1 and PTV2), the conformality of the dose distribution to the targets, and the doses to OARs (rectal wall, bladder wall and prostatic urethra) were evaluated and compared among the dose painting plans using two different MLCs, based on a dose‐volume histogram (DVH) analysis. Furthermore, the conformalities of the dose distribution to the targets were compared with a calculation grid resolution of 2.5×2.5mm,1.25×1.25mm and 5×5mm.

Dose conformalities to the target volumes were calculated using the conformation number (CN), as advocated by van't Riet et al.^(^ ^27^ ^)^ The CN was defined as the product of the fraction of the PTV that received at least 95% of the prescription dose, and the ratio of the volume of the PTV that received at least 95% of the prescription dose to the body volume that received at least 95% of the prescription dose, as shown in the equation:

(1) $$\text{CN}=V_{T,\text{ref}}/V_{T}*V_{T,\text{ref}}/V_{\text{ref}}$$

where $V_{T,\text{ref}}$ is PTV1 or PTV2 that received at least 95% of the prescription dose, $V_{T}$ is PTV1 or PTV2, and $V_{\text{ref}}$ is the body volume that received at least 95% of the prescription dose. CN has values between 0 and 1. A value of 1 represents a reference isodose that covers the planning target volume exactly, without a dose>95% of the prescription dose being irradiated to normal tissues. On the other hand, a value of 0 indicates no dose conformation at all to the target. Dose >95% outside the PTV reduces the conformation number to below 1.

The dose painting plans were designed such that D95 was to be 95% (range from 94.9% to 95.1%) for both PTV1 and PTV2; therefore, the percentage volume receiving at least 95% of the prescription dose (V95) were roughly equal to 95%. As a result, $V_{T,\mathrm{ref}}/V_{T}$ became identical for each plan. Thus, higher CN values represented not only better conformality, but also a better dose distribution and concentration to the target. The CN and doses of the OARs were compared using a paired Student's t‐test. Differences were reported as statistically significant at p<0.05.

## III. RESULTS

### A. Planning outcomes

The average PTV1 and PTV2 volumes were $44.31\;{\text{cm}}^{3}$ (range $38.66–52.13{\text{cm}}^{3}$) and $14.46{\text{cm}}^{3}$ (range $7.64–20.64{\text{cm}}^{3}$) (Table 2).

**Table 2:** Target volume characteristics: PTV1 and PTV2.

*Pat. #*	$\text{PTV}1({\text{cm}}^{3})$	$\text{PTV}2({\text{cm}}^{3})$
1	41.17	7.86
2	48.26	19.95
3	41.87	10.84
4	38.66	14.21
5	39.75	20.64
6	52.13	7.64
7	48.34	20.10

**Figure 1:**
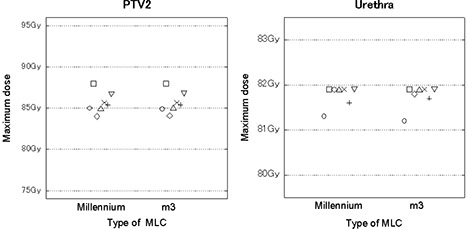
Maximum doses of PTV2 and the urethra in plans using two different multileaf collimators (MLCs).

All of the plans for seven cases with two different MLCs met the goals set in this study. There were no significant differences in D95 for PTV1 and PTV2 among the two different MLCs (Table 3), and the maximum dose to PTV2 was almost identical among the two different MLCs (Fig. 1). In all cases, the maximum dose to the urethra was <82Gy, and the dose distribution was nearly comparable among the two different MLCs (Fig. 1).

**Table 3:** The percentage of the prescription dose covering 95% of the planning target volume (D95), according to the two multileaf collimators (MLCs).

	*Type of MLC*		
	*m3*	*Millennium*	*p*
PTV1 (Prescription dose: 74 Gy)	95.0±0.1	95.0±0.0	0.39
PTV2 (Prescription dose: 84 Gy)	94.9±0.1	94.9±0.1	0.60

Values are expressed as the mean±SD.

### B. Planning comparisons

**Figure 2:**
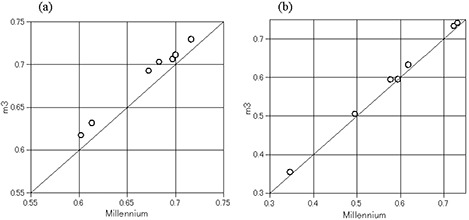
Comparison of the conformation number of PTV1 (a) and PTV2 (b) between the m3 and Millennium plans with a calculation grid resolution of 2.5×2.5mm.

Comparing the CNs for PTV1 between the m3 and the Millennium plans, all of the data points were above a diagonal line, which indicates that the CNs of the Millennium plans were consistently less than those of the m3 plans. Similarly for PTV2, the CNs of the Millennium plans were consistently less than those of the m3 plans (Fig. 2). All of the differences in the CNs for PTV1 and PTV2 were statistically significant (Table 4).

**Table 4:** Comparison of conformation number (CN) values among the two multileaf collimators (MLCs) with different calculation grid size.

	*The Size of Calculation Grid Resolution*	*Type of MLC*		
		*m3*	*Millennium*	*p*
CN for PTV1	1.25×1.25mm	0.68±0.05	0.66±0.04	<0.01
CN for PTV2	1.25×1.25mm	0.59±0.13	0.57±0.13	0.03
CN for PTV1	2.5×2.5mm	0.68±0.04	0.67±0.04	<0.01
CN for PTV2	2.5×2.5mm	0.59±0.12	0.58±0.12	<0.01
CN for PTV1	5×5mm	0.69±0.04	0.68±0.04	<0.01
CN for PTV2	5×5mm	0.61±0.13	0.61±0.13	0.01

Values are expressed as the mean ± SD.

The percentages of the rectal and bladder wall volumes receiving the specified doses were higher with the Millennium plans than with the m3 plans in most cases. But their absolute values were small (Table 5).

**Table 5:** Mean volume (%) of the rectal wall and bladder wall at endpoint doses of 20, 40, 60, and 70 Gy, according to the two multileaf collimators (MLCs).

	*Rectal Wall*			*Bladder Wall*		
*Type of MLC*	*m3*	*Millennium*	*p*	*m3*	*Millennium*	*p*
V20Gy (%)	80.1±6.3	80.7±6.1	<0.01	34.7±13.2	35.5±13.7	0.02
V40Gy (%)	47.8±6.7	48.6±6.7	<0.01	19.6±4.8	20.3±4.9	<0.01
V60Gy (%)	21.6±5.1	22.2±5.2	<0.01	11.7±2.7	12.1±2.7	<0.01
V70Gy (%)	11.1±4.8	11.1±4.9	0.92	5.5±1.9	5.7±1.9	0.03

Values are expressed as the mean ± SD.

Table 4 summarizes the CNs for both PTV1 and PTV2 among two multileaf collimators for three different calculation grid resolutions. The CNs for calculation grid resolution of 5×5mm and 2.5×2.5mm were almost equal or higher than those of 2.5×2.5mm and 1.25×1.25mm, respectively.

**Figure 3:**
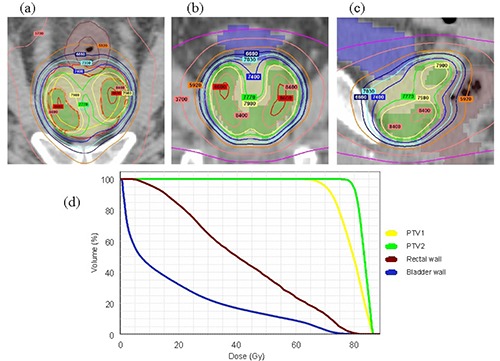
Examples of the dose distribution of intraprostatic dose painting plan and dose volume histogram: (a) axial section; (b) coronal section; (c) saggital section; (d) dose‐volume histogram.

**Figure 4:**
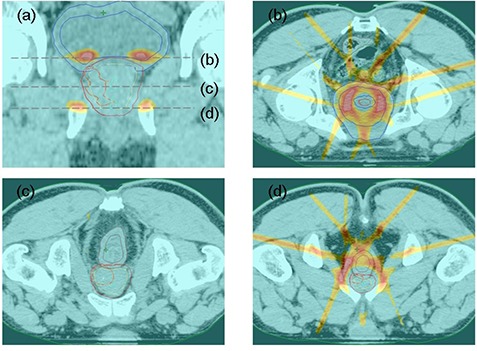
Example of the difference in dose distribution between the m3 and Millennium plans for: (a) coronal section; (b) horizontal section at the level of the base of the prostate; (c) central region of the prostate; (d) apex of the prostate. The area displayed in color has a difference in dose distribution of >2Gy. The area displayed in red represents an area with a difference of >5Gy.

The examples of dose‐volume histogram and dose distributions are shown in Fig. 3, while subtracted dose distributions between the plans created for one case using different MLCs are shown in Fig. 4. In a horizontal section that included the base of the prostate and apex, a difference in dose distribution was found between the m3 and Millennium plans.

## IV. DISCUSSION

The impact of MLC‐W has been reported in several studies, and dosimetric advantages associated with thinner MLCs have been reported for stereotactic radiosurgery and IMRT, mainly in intracranial and head‐and‐neck cancer.^(^ ^20^ ^,^ ^22^ ^,^ ^23^ ^)^ It is expected that MLC‐W would also have an impact on dose distribution in the case of intraprostatic dose painting plans for prostate cancer because the doses to the relatively small targets within the prostate are intensified while retaining all dose constraints to the OARs. To our knowledge, there have been no reports regarding the impact of MLC‐W on dose painting plans for localized prostate cancer using DMLC technique.

In IMRT treatment planning, it is difficult to simply compare different treatment units because of the large degree of freedom in dose delivery with the aid of computer optimization. Therefore, we set the same goals with respect to the targets and OARs, and repeated the optimization until all the goals were satisfied. In the present study, we set the goals that D95 was to be 95% (range 94.9%–95.1%) for both PTV1 and PTV2, and that the maximum dose to PTV2 was to be identical within ±0.1Gy among the different MLC plans. There were only very small differences in target coverage among the plans using the two different MLCs (Table 3 and Fig. 1). Therefore, a plan with a better conformity index is superior in this situation, because the target coverage is basically identical.

The maximum dose to the prostatic urethra was limited to <82Gy (Fig. 1). We did not set strict dose constraints for the rectal wall and bladder wall but limited the doses to the OARs in order to meet the dose‐volume constraints that we currently apply in the clinical setting. Ideally, in comparing the CNs of two different MLCs, our plans should have included strict equivalent doses to the OARs. However, given the difficulty of achieving the dose constraints set for PTV1, PTV2 and the prostatic urethra, it was unrealistic to set equivalent dose distributions to the OARs. Therefore, we set the dose constraints of the rectal wall and urinary bladder wall to meet the limits that we usually use in the clinic. The plan results show that the doses at the rectal wall were significantly lower for thinner MLCs, although the absolute values of the differences were small (Table 5).

The current study indicates that as MLC‐W becomes thinner, the concentricity of the dose improves. However, differences in dose distribution between the m3 and Millennium plans were small. Regions with larger differences in dose distribution were located mainly in the superior and inferior borders of the fields (Fig. 4), which suggests that the MLC‐W can put a direct impact on the dose distribution in the superior and inferior borders of the fields. On the other hand, only small differences were observed in the central part of the prostate, probably because the DMLC method provides for fine resolution of the dose steps along with the driving direction of the leaves. Therefore, if we were to use a multiple static field technique with fewer steps, the difference may become larger.

Only slight differences in the CNs were found among two different MLCs; thus the clinical influence of these differences is unclear. If these differences in conformality were to be spatially dispersed, the clinical impacts may be very small. Very small differences in dose distribution were found, as indicated in Fig. 4. This indicates that replacing a MLC of 5 mm width with one of 3 mm width has a relatively small clinical impact on dose distribution in intraprostatic dose painting plans using the DMLC technique for prostate cancer. Therefore, although a MLC of 3 ‐mm width can physically achieve a slightly better dose distribution, a MLC with a 5 mm width can also be used to achieve the expected dose distribution with this approach.

The volumes of PTV2 were smaller than those of PTV1 (Table 2). On comparing the CNs for PTV1 and PTV2, the CNs for PTV2 were consistently less than those for PTV1, which indicates that the concentricity would become poor. Because PTV2 was defined by adding a 5 mm margin to CTV2 as the DILs, it took various shapes in individual cases. Therefore, the concentricity may decrease if the shapes of PTV2 were intricate even if the volumes were identical. Hence, it may not be appropriate to determine the correlation of the CNs and the volumes of PTV with disregard to the shapes, especially for PTV2. A further study is necessary to consider a relation with the CNs.

The CNs for PTV1 and PTV2 were calculated with a calculation grid resolution of 1.25×1.25mm,2.5×2.5mm and 5×5mm (Table 4). Although there were differences in the CNs with different calculation grid size, the absolute values of the differences were small. In addition, the tendency of the difference between two MLCs was similar among three different calculation grid resolutions. Therefore, we consider that the impact of calculation grid size on the comparison of MLC‐W was small, and hence, would not change clinical significance.

The results of the optimization are very much influenced by the optimization algorithm used in the radiotherapy treatment planning (RTP) system, and the results can vary when plans are created with other RTP systems. In addition, treatment machines and delivery techniques can have a large effect on the achievable dose distribution. Therefore, the results of this study may not be applicable to other RTP systems or treatment units.

In the clinical application of this approach, appropriate PTV to CTV (gross target volume) margins should be properly applied. In the present study, we used only a 5 mm margin to create PTV2. We believe that image‐guided approaches such as implanted gold marker‐based or CT‐based error reductions should be used to ensure that the planned treatment doses are actually delivered.^(^ ^28^ ^)^

## V. CONCLUSIONS

The planning goals set in the present intraprostatic dose painting planning protocol for localized prostate cancer were achieved using two different types of MLCs. However, dosimetric advantages associated with smaller leaves were observed in terms of the conformity of the prescribed dose to the target, with a small but significant reduction in the rectal dose. When the DMLC technique is applied in this approach, a MLC with a 5 mm width may be sufficient, because any dosimetric disadvantage compared with a 3 mm‐width MLC was relatively small.

## ACKNOWLEDGEMENTS

This work was partially supported by Grants‐in‐Aid for Scientific Research from the Ministry of Education, Culture, Sports, Science, and Technology (20229009) and Grants‐in‐Aid for Scientific Research on Priority Areas Cancer from the Ministry of Education, Culture, Sports, Science, and Technology (17016036).
